# Mechismo: predicting the mechanistic impact of mutations and modifications on molecular interactions

**DOI:** 10.1093/nar/gku1094

**Published:** 2014-11-11

**Authors:** Matthew J. Betts, Qianhao Lu, YingYing Jiang, Armin Drusko, Oliver Wichmann, Mathias Utz, Ilse A. Valtierra-Gutiérrez, Matthias Schlesner, Natalie Jaeger, David T. Jones, Stefan Pfister, Peter Lichter, Roland Eils, Reiner Siebert, Peer Bork, Gordana Apic, Anne-Claude Gavin, Robert B. Russell

**Affiliations:** 1 Cell Networks, University of Heidelberg, Im Neuenheimer Feld 267, 69120 Heidelberg, Germany; 2 Bioquant, University of Heidelberg, Im Neuenheimer Feld 267, 69120 Heidelberg, Germany; 3 Deutsches Krebsforschungszentrum, Im Neuenheimer Feld 280, 69120 Heidelberg, Germany; 4 Department for Bioinformatics and Functional Genomics, Institute for Pharmacy and Molecular Biotechnology (IPMB), University of Heidelberg, Heidelberg, Germany; 5 Institut für Humangenetik, Universitätsklinikum Schleswig-Holstein, Christian-Albrechts-Universität zu Kiel, Arnold Heller Straße 3, 24105 Kiel, Germany; 6 EMBL, Meyerhofstrasse 1, 69117 Heidelberg, Germany; 7 Cambridge Cell Networks Ltd, St John's Innovation Centre, Cowley Road, CB3 0WS, Cambridge, UK

## Abstract

Systematic interrogation of mutation or protein modification data is important to identify sites with functional consequences and to deduce global consequences from large data sets. Mechismo (mechismo.russellab.org) enables simultaneous consideration of thousands of 3D structures and biomolecular interactions to predict rapidly mechanistic consequences for mutations and modifications. As useful functional information often only comes from homologous proteins, we benchmarked the accuracy of predictions as a function of protein/structure sequence similarity, which permits the use of relatively weak sequence similarities with an appropriate confidence measure. For protein–protein, protein–nucleic acid and a subset of protein–chemical interactions, we also developed and benchmarked a measure of whether modifications are likely to enhance or diminish the interactions, which can assist the detection of modifications with specific effects. Analysis of high-throughput sequencing data shows that the approach can identify interesting differences between cancers, and application to proteomics data finds potential mechanistic insights for how post-translational modifications can alter biomolecular interactions.

## INTRODUCTION

High-throughput sequencing (HTS) has led to the systematic identification of thousands of protein variants (1) from which the aim is to identify those most likely to impact biological systems or cause disease. Advances in proteomics have similarly produced data sets of thousands of post-translational modifications (PTMs) (2) also aiming to find those of biomedical consequence. These are just two examples of the wider trend in life science research where data generation is often faster than interpretation, making tools for aiding the ranking and analysis of such findings of increasing importance.

The current flood of variant and modification data is concurrent with a growing set of protein 3D structures and interactions. Virtually all protein domains now have at least one representative structure, and the number of interactions for which structures are known or modelled grows continuously (3–7). Intense interaction discovery efforts also provide an increasingly complete set of biomolecular interactions (e.g. (8,9)) and there are now tens of thousands of interactions known for most of the major model organisms.

To study even a single residue change in terms of potential functional impacts can require simultaneous consideration of dozens of 3D structures and interactions, and almost invariably requires consideration of homologous proteins; even if a structure of a particular protein is available, functional insights might come only from one with lower sequence similarity that is nevertheless bound to a relevant ligand. To study sets of thousands of protein changes accordingly requires the integration of a vast set of information. Ideally, one would model each change in every available biomolecular interaction context, but to do so would be time-consuming and unfruitful owing to the small likelihood that any one of the thousands of models required would alone be functionally informative. Moreover, comparative modelling at low sequence identities is difficult and prone to inaccuracies, and often simple inspections of an alignment with a template 3D structure are as effective as models.

Several efforts have been made to bridge interactomes and structures (e.g. (4,10–13)) though these have only secondarily addressed protein changes related to mutations or PTMs. Resources such as Interactome3D (4) and 3DID (14) provide an excellent view into the putative structures and molecular mechanism of protein–protein interactions, but do not readily allow the user to consider specific sets of positional changes. Modelling resources, such as ModBase (3) or the SwissModel repository (15), provide millions of pre-computed structures that allow the study of mutations in any individual structure, but models are normally only in one specific context (i.e. one specific template) and often lack functionally informative bound partner proteins, small molecules or nucleic acids, which could very well be only in poorer quality structural templates not used in the modelling processes that are normally aimed at individual model quality and not necessarily functional interpretation.

There are also many methods to predict deleterious mutations, which typically consider protein sequence conservation and/or properties from individual protein structures (or homologues) to estimate deleteriousness (e.g. (16–20)). Newer approaches focus more specifically on aspects of protein function as revealed by sequence conservation patterns (18), though none are designed for detailed analysis of the molecular mechanism of mutations or modifications in the context of known biomolecular interactions. Nevertheless, there is growing evidence that these interactions and 3D interfaces could improve predictions (e.g. (21–24)), in line with the many instances of mutations known specifically to modulate particular interfaces. For instance, Apert syndrome, characterized by skull malformation, syndactyly and mental deficiencies, is caused by mutations in the fibroblast growth factor receptor 2 (FGFR2) that selectively increase the affinity for FGF2 (25). Missense mutations in the DNA methyltransferase DNMT3B, implicated in immunodeficiency, centromeric instability, facial anomalies syndrome, affect both the catalytic site and an N-terminal PWWP domain, involved in protein–protein interactions (26). Mutations are also known to prevent the assembly of functional multi-protein complexes, such as those in the RFXANK gene, implicated in bare lymphocyte syndrome (27), that hamper its ability to assemble a regulatory factor X complex required for the expression of MHC class II genes.

To help predict and understand the impact of mutations or modifications on protein function, we present here a new online resource Mechismo (Mechanistic Interpretation of Structural Modifications) that identifies residue changes (mutations or modifications), from uploaded sets of thousands, likely to have functional consequences by affecting interactions with proteins, small molecules or nucleic acids. To predict the functional impact of a residue change, the method makes use of all possible homologous structures and all available protein–protein interactions and provides an easy interface for non-experts to access a vast and complicated underlying data set.

## MATERIALS AND METHODS

### Data sources and defining 3D interfaces

We extracted sequence fragments from 3D structures (28) as either entire chains or domains defined in the Structural Classification of Proteins (SCOP) database (29). We did not restrict structures on the basis of resolution or other quality measures as we do not want to exclude lower quality that might nevertheless contain bound molecules absent in better quality structures (though we did do this for parameter calculations; see below). We defined 3D protein–protein interactions as pairs of fragments with at least 30 pairs of residues having side-chain atoms within 5Å in biological assemblies (ignoring crystal-contacts). We grouped interactions when they involved instances of fragments with identical sequences, and when pairs of residues in contact were the same, and selected one representative 3D interaction for subsequent use.

We defined interactions between proteins and small molecules or DNA/RNA by identifying side-chains within 5Å of atoms excluding solvents commonly used in crystallization and experimental modifications (30). We also defined 57 classes of chemicals of known structure by all-against-all fingerprints searches (31), manual inspection and cross-referencing to DrugBank (32) to capture the major types of chemicals in the database (e.g. metals, nucleotides, amino acids), metabolites, known drugs or compounds very similar to them and larger categories for other compounds (Organic, Inorganic, Organometallic). These classes are listed in Supplementary Table S1.

We compared UniProt (33) sequences to the representatives above using Basic Local Alignment Search Tool (BLAST) (34) considering matches with *E*-value threshold of 0.0001 and storing the best match for each position in the UniProt sequence. Best matches (by sequence identity) of pairs of UniProt sequences from the same species to representative 3D interactions were selected as models for the interaction. For all sequences we also defined protein domains by significant HMMscan matches to Pfam domains (35), and intrinsically disordered residues as those where the mean IUPred (36) long disorder of the matching fragment residue over a sliding window of 11 residues was ≥0.5. We also used the domain graphics library from Pfam to display domain diagrams.

### Protein interaction data

We merged protein–protein interactions from four major primary protein interaction databases: BIND, BioGRID, IntAct and MINT (33,37–41) making all of them centric on Uniprot by accession mapping. The resulting data set contains 845 944 unique biophysical/biochemical interactions from 1 058 604 interaction records and 49 285 publications. We used UniRef (33) to group interactions at different degrees of sequence similarity and to map interactions to orthologues. To define direct-physical interactions we used the MI Ontology (42) of detection methods, excluding mass-spectrometry identified complexes from this set. We defined high-throughput experiments as those having 300 or more interactions in a single publication, and high-quality interactions as those detected by two or more distinct publications or detection methods. Note that all interactions are stored in the database, though through the interface users can restrict interactions to only those that are direct-physical, high-quality or to select the degree to which homology (Uniref100, Uniref90 or Uniref50) is used to infer interactions between organisms. For the analyses in the text, all possibly physical interactions between proteins are considered, provided they have at least one interface of known 3D structure on which to model them.

### HTS and phosphoproteomic data sets

We downloaded specific tables from the original papers for Medulloblastoma (43), Pancreatic Cancer (44) sequencing and *Escherichia coli* phosphoproteomics studies (45) and mapped both to sequencing data using Uniprot (33) accession matching or genomic coordinates via Ensembl (46). For the phosphoproteomics data we considered all positions if there was an ambiguity about site assignment (i.e. multiple phosphorylateable residues in the same peptide), and for mutations we only considered non-synonymous changes, ignoring frame-shifts or stop-gains.

### Measures of prediction confidence

We defined a gold-standard positive set of sites as residues from model proteomes in contact with any interacting molecule (protein, chemical or DNA/RNA) with a sequence to structure similarity of ≥90%, and a negative set as those residues not in the positives and predicted to interact with molecules using templates structures with ≤20% identity. The negative set includes some positives as these can still occur at low similarities, thus making confidence measures conservative. We then computed the proportion of sites recovered using model proteome sequences sharing sequence identities between 20–90% with structures, computing true-positive and false-positive rates (TPR and FPR) for each type of interaction (protein, chemical or DNA/RNA) and sequence identity. Note that these values are for whether a changed amino acid is in contact with another molecule; the accuracy of predicting directions (i.e. enhancing or diminishing) is described in the next section. The entire data set is available from mechismo.russelllab.org.

### Assessing whether changes enhance or diminish interactions

We computed interface pair-potentials (47) for residues to be in contact with other residues, chemicals or DNA/RNA by considering a non-redundant set of individual interfaces defined initially using high-quality (in terms of resolution, refinement, etc.) representatives (48) of SCOP domains, though by keeping only one instance of proteins in any Uniref50 group. To deal with phosphorylated Ser/Thr/Tyr (pS, pT, pY) and acetylated Lys (aK) residues we also added interaction structures containing these residues (400, 612, 501 and 114, respectively) that we then made non-redundant by the same process. For protein–protein interactions, we defined side-chain contacts as van der Waals (VDW, C-C or C-S within 4.5 angstroms) or electrostatic (including Hbond; N or O atoms within 5.5 angstroms) and defined meaningful contacts as those involving the functional aspects of side-chains (VDW: A, C, F, G, I, L, M, P, V, W, Y, pY, aK; Electrostatic: D, E, H, K, N, Q, R, S, T, W, Y, pS,pT, pY, aK). We made no such distinction in side-chain atom type for DNA or chemical interactions, requiring only side-chain contacts with any non-protein atom from the particular molecule class. The distance thresholds were assigned according to previous studies of protein interactions (47,49), and are essentially the upper limits for electrostatic or hydrogen-bonding distances (50) and twice the Carbon van der Waals radius (3.8 angstroms) plus a fudge factor (0.7 angstroms) to allow for resolution issues. Matrices generated with simpler cutoffs (e.g. all-atom 4.0, 4.5, 5.0, 5.5 and 6.0 angstroms) showed poorer reproduction of expected groupings of the amino acids (negative: D/E/Sp/Tp/Yp; positive: R/K; aromatic: Y/W/H; hydrophobic: I/L/M/V/F; small-hydroxyl S/T; amide Q/N; small P/A/C) compared to the matrix derived with the first parameters above. The small size of the benchmark (below) makes it difficult to perform a systematic analysis of different thresholds; all sets tested gave similar results.

For each type of interaction (protein, DNA/RNA or chemicals) we defined the expected frequency (*f*_exp_) as the product of interface residue frequencies (protein–protein interactions), or as the frequency of the residue in proteins binding to DNA/RNA or particular chemical classes (i.e. a molar-fraction random model (47)). For each residue type we then measured the observed frequency (*f*_obs_) to be in contact with a particular residue, chemical or DNA/RNA, and computed:

\begin{equation*} \begin{array}{*{20}c} {{IPP}_{aa} = \log \left( {\frac{{f{\rm obs}}}{{f{\rm exp}}}} \right)\quad {IE}_{{\rm change}} } { = \sum\limits_{{\rm all}\;{\rm res}\;{\rm conts}} {\left( {{IPP}_{{\rm change}} - {IPP}_{{\rm wt}} } \right)} } \\ \end{array} \end{equation*}

where *IPP*_aa_ is the value for a particular pair (with *IPP*_wt_ as the value for the wild-type and *IPP*_change_ as the value for the altered residue).

For the majority of chemical classes, there was insignificant enrichment or depletion of residues to make the measures above useful (i.e. log odds <1), so these were excluded. This is likely due to the failure of this generic approach to capture the diversity in molecules with varied properties (e.g. adenosine triphosphate (ATP) has both hydrophobic and negatively charged moieties). Nevertheless, we obtained satisfactory *IPP*_aa_ values for protein–protein, protein–DNA/RNA and interactions with Zn, Mg, Fe, Mn, Cu ions. Updates will include similar parameters for repetitive chemical moieties, such as phosphate, sulphate and carboxylate. Note that *IPP*_aa_ values shown in the figures are multiplied by 10 and capped at 9 for clarity (11 values).

**Figure 1.**
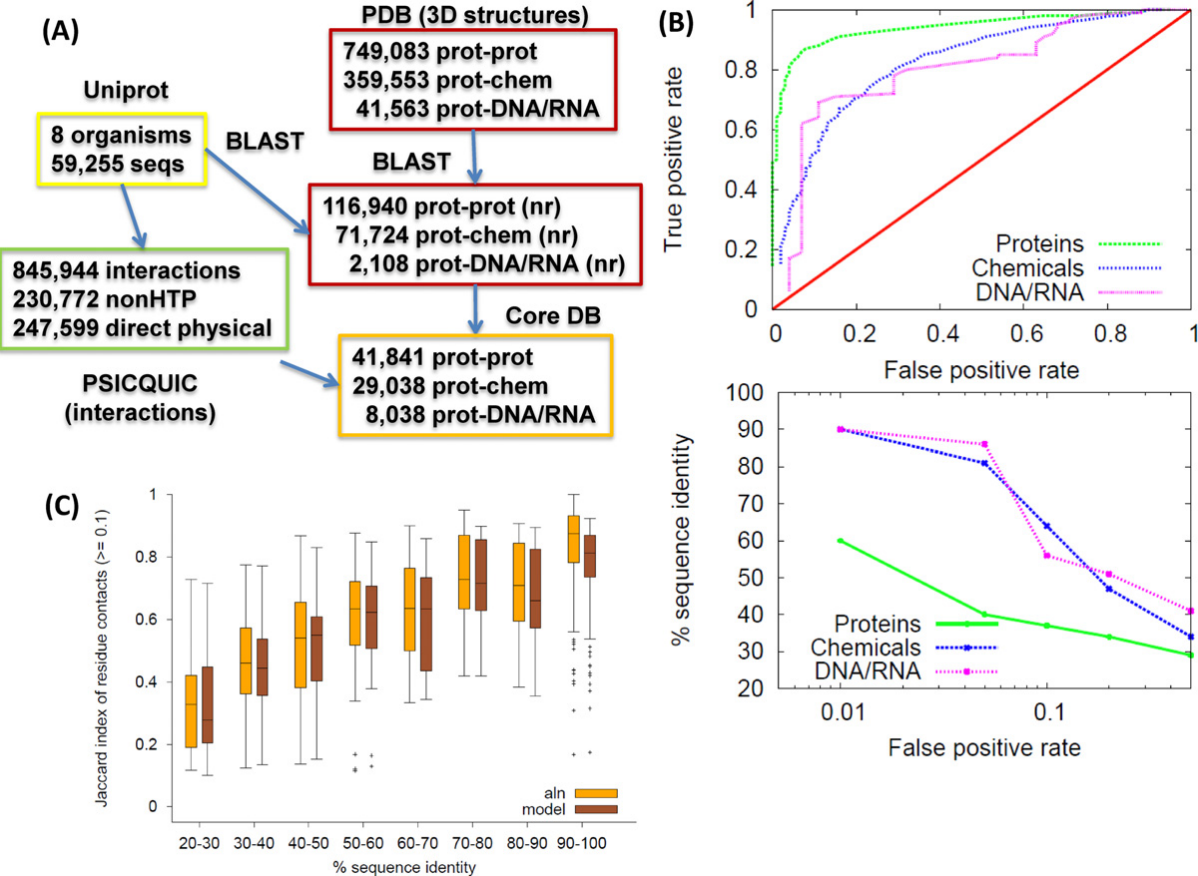
(**A**) Schematic of the data sources and pipeline. Numbers from the PDB (3D structures) at the top denote the complete set of interactions of each type, the second list (nr) refer to those that are non-redundant when grouping identical sequences. The final numbers (Mechismo Core DB) are those with the least stringent filtering criteria, though still requiring some sequence similarity and knowledge of protein–protein interactions (weakest). The totals without any filtering are 2 952 035 protein–protein, 51 182 protein–chemical and 13 186 protein–DNA/RNA, which in practice are only useful with species with small genomes and/or lacking structure data (e.g. yeast and bacterial species). (**B**) ROC curves (top) for predicting sites at protein, chemical and DNA/RNA interfaces, plotted by modifying the sequence identity threshold and reporting FPR/TPR. The associated values of sequence identity for key FPRs derived from this plot are shown in the plot below. (**C**) Box-plots (Tukey) showing contacts preserved (Jaccard index) versus sequence identity for protein–protein interactions where contacts are either inferred using an alignment to a 3D template (aln) or taken from a model of the interface constructed by homology modelling. For the box-plots we ignored datapoints where the Jaccard index was <0.1, as these denote different interfaces. Equivalent plots for protein–chemical and protein–DNA/RNA are shown in Supplementary Figure S1.

**Figure 2.**
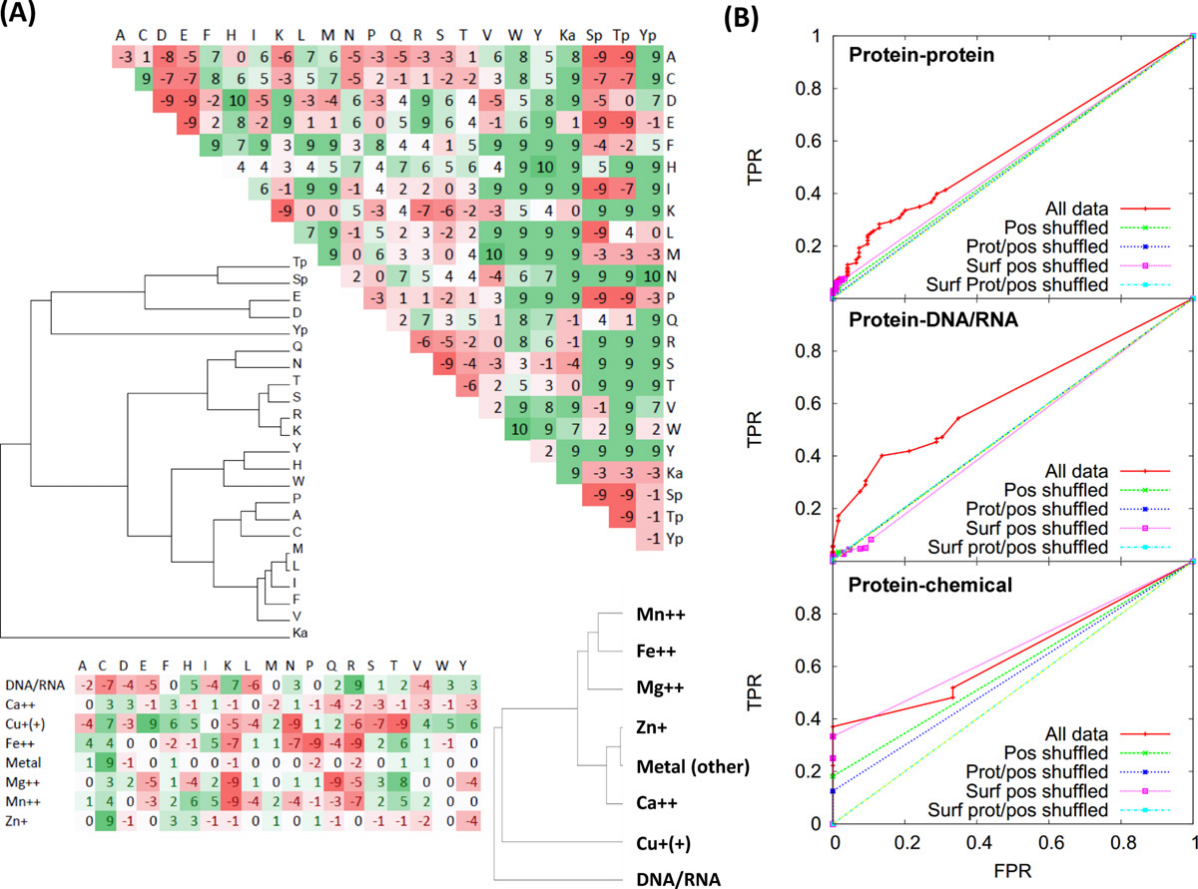
(**A**) Log-odds scores for amino acid side-chains interacting with other side-chains (top) and DNA/RNA and chemicals for which appropriate parameters are available (bottom). Values are multiplied by 10 and stripped of decimals for clarity, and are coloured red if unfavourable and green if favourable, with darker colours indicating stronger values. Modified amino acids are given as: Ka, acetyllysine; Sp/Tp/Yp, phospho-serine/threonine/tyrosine. Note that this is not a mutation or substitution matrix, but a measure of residue–residue interactions. The dendrograms show means clustered groups using distances between amino acids/molecules calculated by summing the absolute differences between matrix values. (**B**) ROC curves showing how accurate the direction of interaction effect is predicted based on a data set human mutations with annotated in Uniprot for disabling/enabling effects on protein, chemical or DNA/RNA interactions. ‘All data’ denotes the data set, with various shuffled data sets also shown: Pos denotes different positions in the same protein, Prot/pos denotes different proteins and positions and Surf denotes where accessibility values are maintained between the original data set and the shuffle.

*IE*_change_ is the score associated with mutations or modifications, with contacts involving the mutated/modified residue being presumed to be the same as the original residue. Note that we also presume the same contacts when the template structure differs from the original sequence. High positive values indicate modifications predicted to favour an interaction (e.g. changing Asp interacting with two Phe residues to Leu gives a value of 2.4), whereas negative values would be predicted to disfavour it (e.g. changing Arg interacting with two Glu residues to Cys gives a value of −3.8).

We defined the benchmark set by extracting all human mutations from Uniprot (MUTAGEN and VARIANT) marked features and their associated text. From these 135 275 mutations we then looked for descriptions containing ‘bind*’ or ‘interact*’ leaving 6283, which we inspected manually, labelling them whether they disabled/enabled (the positives for receiver operator characteristic (ROC) analysis) or had no effect (negatives) on the interaction with particular proteins, complexes, protein classes, chemicals, chemical classes, DNA or RNA. For sites targeting common complexes or classes (actin, clathrin, collagen, G-proteins, Histone H3, importin alpha/beta, microtubules, nucleoporin, ribosome, RNA polymerase II and tubulin), we allowed the possibility that the mutation could affect interactions with any of the proteins in these human complexes or classes. This expansion plus the fact that many sites described effects on multiple individual proteins (e.g. abolishes interactions with A, B and C, but has no effect on D binding), led to a final set of 12 527 mutation/interactions pairs.

We defined a total positional impact (Mechismo) score as the sum of the highest absolute *IE*_change_ values plus 1 for each protein, chemical or DNA/RNA site found. This addition allows for incalculable *IE*_change_ values in chemicals and gives non-zero values to all sites at any functional interfaces. We also defined a total protein impact score as the sum of these values for all sites in a protein from a particular data set.

### Comparison with protein comparative modelling

We compared mechismo (i.e. alignments with structures) to full protein modelling by first selecting a random sample of 4146 non-redundant interfaces (205 protein–protein, 2660 protein–chemical, 1281 protein–DNA/RNA); for chemicals we defined redundancy according to the chemical classes in Supplementary Table S1 (instead of discrete chemicals). For each of these we found all other interfaces (i.e. templates) involving homologous proteins or similar chemicals/DNA/RNA and grouped these according to sequence identity (bins at intervals of 10%) to the selected interface. We then selected one interface from each bin and used the alignment from the database to construct models using the Automodel feature in MODELLER (51). We then calculated the proportion of interface contacts in the selected structure that were reproduced using either the modelled structure (model) or the alignment to the template (aln) as a Jaccard index (the intersection divided by the union).

### Shuffling data sets and assessing significance

We created shuffled data sets by both moving particular modifications to random positions in the same protein (sites only) or by moving them to a random position in a randomly selected protein from the same data set (proteins and sites). In doing so we did not permit the original site to be chosen, which for rare amino acids in shorter proteins could be problematic for the sites-only data set (e.g. if a protein of 30 amino acids has only a single Arginine that is mutated). For certain contexts (e.g. PTMs) it is worthwhile to consider sets with a similar distribution of surface/buried residues. We constructed this set from the entire data set, but taking the best 3D template for any proteome segment (any significant BLAST match as above) and computing NACCESS (52) accessibilities, computing relative accessibilities to define classes of buried (≤5%), intermediate (>5, ≤25%) and exposed (>25%) and an additional class for instances when no 3D data was available. When randomizing, we required the same distribution across the four classes. We refer to these as surface-biased (proteins and) sites shuffled data sets. To assess the significance of the overlap, we used a two-sided Fisher's exact test, considering the total number of sites in the data set and the sites of a particular class (e.g. protein–protein, protein–chemical, protein–DNA/RNA) in the two sets being compared. Note that these sets are not used as negatives in any of the ROC analyses: negatives are defined in different ways, but are never shuffled data sets.

### Studying deleterious mutation prediction methods

We used ConDel (20) to extract PolyPhen2 (16), SIFT (17) and MutationAssesor (18) predictions for 19 800 deleterious and 24 082 neutral mutations collated from multiple sources (53). Differences in genome versions and accessions meant that only 14 837 deleterious and 16 068 neutral mutations were available for all three methods.

### Open reading frame cloning

For the interactions with mutations discussed for RhoA (see Results and Discussion), we obtained 11 clones as sequence optimized synthetic DNA from commercial suppliers (LifeTechnologies/IDT), in terms of codon optimization for expression in *Saccharomyces cerevisiae*, GC-content and restriction sites. The sequences encoding the proteins were flanked by attb-Gateway sites (Invitrogen) for further cloning. All constructs were shuttled into the Donor vector pDONR221 by Gateway BP-reaction and subsequently by LR-reaction into the Y2H bait and prey vectors pDEST32 and pDEST22, respectively, for the Yeast two-Hybrid experiments. All constructs were sequence verified.

### Yeast two-hybrid assays

We performed two-hybrid assays following an altered ‘Testing specific Two-Hybird interaction’ protocol of the ProQuest™ Two-Hybrid System Handbook (Invitrogen). Briefly, we co-transformed all interaction pairs (Interactor/RhoA) into yeast strain MaV203 (Invitrogen, MaV203 Competent Yeast Cells, Library Scale cat# 11281–011). Colonies from each transformation were grown on 15-cm plates of synthetic complete media lacking leucine and tryptophan (Sc-Leu-Trp). After 2–3 days we picked three individual colonies of each transformation and suspended them in 100 μl autoclaved saline in a 96-well plate. To achieve a uniform cell density, we transferred 10 μl of the suspension to 150 μl SC-Leu-Trp medium in another 96-well plate. These cultures were grown overnight at 30°C in an incubator without shaking to reach saturation. We re-suspended the cultures by vigorous shaking followed by replication with a 96-needle replicator onto rectangular SC-Leu-Trp-His readout agar plates containing different concentrations (5, 10, 25, 50 and 100 mM) of the inhibitor 3-aminotriazol. We interpreted phenotypes 2–5 days after plating.

## RESULTS AND DISCUSSION

### The database

The core database currently combines 86k 3D structures (including 117k protein–protein, 72k protein–chemical and 2k protein–DNA/RNA non-redundant interfaces) cross-referenced to 846k protein–protein interactions and 60k proteins in eight model organisms (Figure 1A) with 59k sequences and 30 million residues (Figure 1A). Coverage is extensive in terms of the proportions of proteins making at least one contact with proteins, chemicals or nucleic acids. Overall, 51.0% of all proteins have at least one known or potential protein–protein interface, 45.7% have at least one protein–chemical interface and 13.6% a protein–DNA/RNA interface, with differences across the species (Supplementary Table S2) probably reflecting biases in species used for structure determination. The fraction of residues lying within functionally informative structures (i.e. structural matched regions that bind other proteins, chemical or nucleic acids) is 23.2% for protein–protein, 18.5% protein–chemical and 4.5% protein–DNA/RNA and 33.9% when all three are combined (N.B.: this is not a summation as several regions bind more than one molecule type). This combined residue coverage ranges from 29.3% in *S. cerevisae* to 55.2% in *E. coli.*

### User data, parameters and interface

Uploaded proteins and changes are mapped to the database and 3D interfaces filtered by thresholds for sequence/structure similarity and for interactions to be used. Users can modify the stringency of data considered in terms of the lowest sequence similarity and the nature of protein–protein interactions (e.g. higher quality, low-throughput or direct physical interactions). In all the examples discussed below, we used the least stringent setting: considering protein–protein interactions with any published evidence of a physical association between proteins, and any sequence identity to statistically significantly matched structures (‘low stringency’ on the website). Increasing stringency can be useful when considering proteins with substantial structural and/or interaction partners (e.g. GTPases, P53, etc.).

After job completion, the user is presented with overall totals for proteins/positions, a network view of interactions and changes, and summary tables for proteins and changes. Additional pages describe individual proteins/changes, and still others describe structural matches, giving alignments, domain diagrams, views of changed positions in structures and references describing interactions. For simplicity and speed, we do not construct or download homology models, but present alignments beside matched structures, highlighting differences between the original sequence and the structure that might affect prediction accuracy (see below).

### Assessing accuracy of individual functional site predictions

Even if the structure of a particular protein is known, those with interacting proteins, chemicals or DNA/RNA are often only available for homologues with lower sequence identities. To use such a diversity of structures requires measures of prediction confidence as a function of sequence similarity, which we devised by defining gold-standard positives and negatives and testing the ability of sequence/structure matches with different degrees of identity to predict them (Materials and Methods).

Residues at functional interfaces in structures identical or nearly identical to proteins from model organisms are considered to be positives, and those from structures with very low sequence similarity (but not in the positives) are defined as negatives. The negatives likely contain many positives that are only identified at very low sequence identities, making the benchmark conservative. All other structures (20–90% sequence identity) are then used to predict all positive and negative sites. ROC analysis gives FPRs that provide confidence measures when predicting a site for each type of interaction at any sequence identity (Figure 1B). These values vary across the different molecule types, with protein–protein interactions having high-confidence at as low as 40% identity, but protein–DNA/RNA interactions requiring a higher threshold (56%) to reach the same degree of confidence.

A natural question is whether it would be more accurate to construct homology models of all proteins, as opposed to interrogating an alignment beside a 3D structure. We tested this by sampling 4000 3D interfaces of all three types, modelling (51) them on homologous interfaces at a range of sequence identities and comparing the interface contacts between the model/template to the original structure. The results suggest that there is little difference between homology models constructed this way and the simpler alignment/template strategy (Figure 1C; Supplementary Figure S1). More careful modelling strategies might give an improvement, but these are not practical to run over many thousands of mutations or modifications.

### Assessing where modifications enhance or diminish interactions

Mutations and modifications can either enhance or diminish biomolecular interactions. To predict such effects, we used pair-potentials (54) for protein interaction interfaces developed previously (47) and extended to consider phosphorylated/acetylated residues, protein–chemical and protein–DNA/RNA interactions (Materials and Methods; Figure 2A). The potentials are log odds, with high positive numbers indicating a relationship (e.g. residue–residue, residue–DNA, residue–chemical) that is seen more often than expected from the abundance of amino acids at interfaces and high negative numbers indicating the opposite. We used these values to define *IE*_change_ (Materials and Methods) that measures the effect of changing an amino acid at an interaction interface by computing the difference between these log odds scores for the original residue and those for the mutation or modification.

In general, the parameters for residue pairs (Figure 2A, top) agree broadly with those computed previously for protein–protein interfaces (55) though to our knowledge no set has to date included the modified amino acids. As expected, phosphorylated Ser/Thr (Sp/Tp) are similar to negatively charged amino acids in their preferences, with tyrosine phosphate (Yp) varying between negative and aromatic residues. Interestingly, acetylated Lysine (Ka), though generally an outlier from all amino acids, appears to prefer hydrophobic/aromatic environments, in contrast to being akin to Glutamine as is often considered to be the best natural substituent (e.g. (56,57)). Interestingly, this agrees with recent observations that Methionine mutations can mimick Ka in cancers (58). Parameters for DNA/RNA contacts are also as expected (Figure 2A, bottom), with positive residues favoured, and negative or most hydrophobic residues disfavoured, with aromatic or polar residues being close to neutral. Contacts to metals differ, also as expected, for instance with the classic tetrahedral coordinating Cys/His favouring Zn^+^, Asp (but not Glu) favouring Ca^++^ and small polar residues (Ser/Thr) favouring Mg^++^/Mn^++^ (59), though there are certain differences that inspection suggests are likely to do with our oversimplistic model that does not consider ionic charge or coordination shells, which would not necessarily be accurately modelled at low sequence identities to structures.

We tested the effectiveness of these values by investigating 5127 human site-directed mutations and 805 disease variants within Uniprot that were annotated to have an effect on interaction, binding or affinity with other molecules. After manual editing, 4070 site-directed (mutagen) and 508 disease variants could be mapped to particular proteins, 559/164 to specific chemicals and 498/133 to DNA or RNA interactions. As expected, the data are heavily biased towards disabling rather than enabling or neutral effects on interactions: 79.0% (4051 mutagen and 634 disease variants) are disabling, 3.2% (143 and 39) enabling and 17.8% (926 and 132) are neutral. Many mutations are annotated as affecting multiple molecules, making a final set of 12 527 mutation/molecule pairs (with some redundancy owing to molecule descriptions such as ‘actin’ or ‘RNA polymerase II’), of which about 10% (1257) could be matched to a protein structure for which there were known/predicted structures related to the interaction observed (1038 mutagen and 219 variants).

ROC curves (Figure 2B) show that these scores are able to assess the impact in terms of direction with moderate sensitivity and specificity in contrast to randomly shuffled data where either the position of the mutation or both the position and the protein were selected at random. We did not see any appreciable improvements when exploring different ranges of sequence identity, though when doing so data become sparse (i.e. too few examples remain in the benchmark). Note, in addition, that for protein–chemical interactions we had only five negatives which accounts for the rather abrupt appearance at the bottom plot in Figure 2B.

To score and rank changes we defined an overall Mechismo score for individual sites as the sum of highest absolute *IE*_change_ values for protein, chemical and nucleic acid effects (considering only the highest values for each type of interaction when multiple molecules contacted any single site), and an overall score for proteins/genes as the sum of these values for all changes in each protein. This allows the most functionally impacted sites and proteins from large data sets to be identified.

### Searching for edgetic effects in cancer mutations

**Figure 3.**
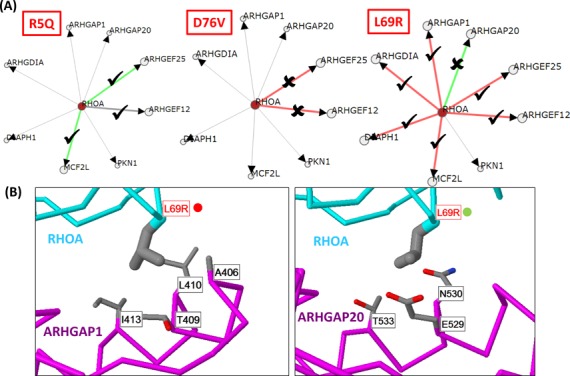
(**A**) Network of RhoA and interaction partners showing the predicted effect of each mutation on a selection of interaction partners with structures sharing very high sequence identities with the human protein. Green lines show interactions where RhoA mutations are predicted to enhance the interaction; red lines where they diminish. Proteins linked with thin lines are those that interact with RhoA via an interface of known structure that does not involve the mutation. Tick/cross marks denote whether the proposed effect was observed in the two-hybrid tests. (**B**) Structures of RhoA mutation L69R in contact with three interactors. Proteins are shown as C-alpha trace with residue side-chains shown as wireframe (carbon = grey; oxygen = red; nitrogen = blue). Red labels show the location of the mutated RhoA residue; black those with which it is interacting on the other protein. Red circles indicate a disabling prediction, green an enabling one.

The values above provide the means both to identify *edgetic* effects (60) that impact a single edge in a network, and to judge whether mutations might be affecting networks by shifting the balance of affinities for different partners, rather than completely destroying a particular interaction. For example, in a data set for Burkitt's Lymphoma (61) we identified a series of mutations in the RhoA (62) GTPase that were predicted by detailed modelling efforts to shift the affinities for various effector proteins (GAPs, GEFs, etc.). The method very rapidly reproduces the original findings (Figure 3) showing a diversity of effects for the three mutations on different RhoA regulators, including the observation that L69R could potentially enable an interaction with ARHGAP20 while disabling the others. The structures show that this residue normally resides in a hydrophobic pocket in all partners apart from ARHGAP20 where it sits next to E529, N530 and T533. The changed residue Arginine could possibly form a favourable salt-bridge with a glutamate and make additional favourably polar contacts with the others (Figure 3B). We tested the 12 RhoA mutation/interaction pairs highlighted in Figure 3A using the two-hybrid system for whether or not they had the predicted effect on particular interactions (Supplementary Table S3; Supplementary Figure S2). We found that 9/12 had the predicted effect on the interface. Specifically, for these we found the mutations to have a weaker signal in the assay when predicted to be disabling when compared to the wild-type; enabling mutations did not show a clear increase in interaction (as might be expected given the coarseness of the assay), though growth similar to wild-type was taken to be a success.

### Application to HTS data sets

To demonstrate the use of this approach on HTS data we considered two cancer data sets. The first is a set of 641 non-synonymous mutations in 569 proteins identified in Medulloblastoma tumors by a combination of exome and whole genome sequencing (43). After integrating 2368 structures and 17 871 interactions, the method identifies 92 sites with predicted functional consequences. The second includes 2850 mutations in 1712 proteins identified in pancreatic cancers by exome sequencing (44), where the method identifies 212 functionally relevant sites. These two data sets are similar in that they both show enrichment for protein–chemical interactions when compared to shuffled data sets (significant at *P* < 0.05 or *P* < 0.01 for all apart from surface/positions-only; Supplementary Table S4). Both are also enriched in mutations at protein and nucleic acid binding sites relative to shuffled sets, but the differences are not significant.

**Figure 4.**
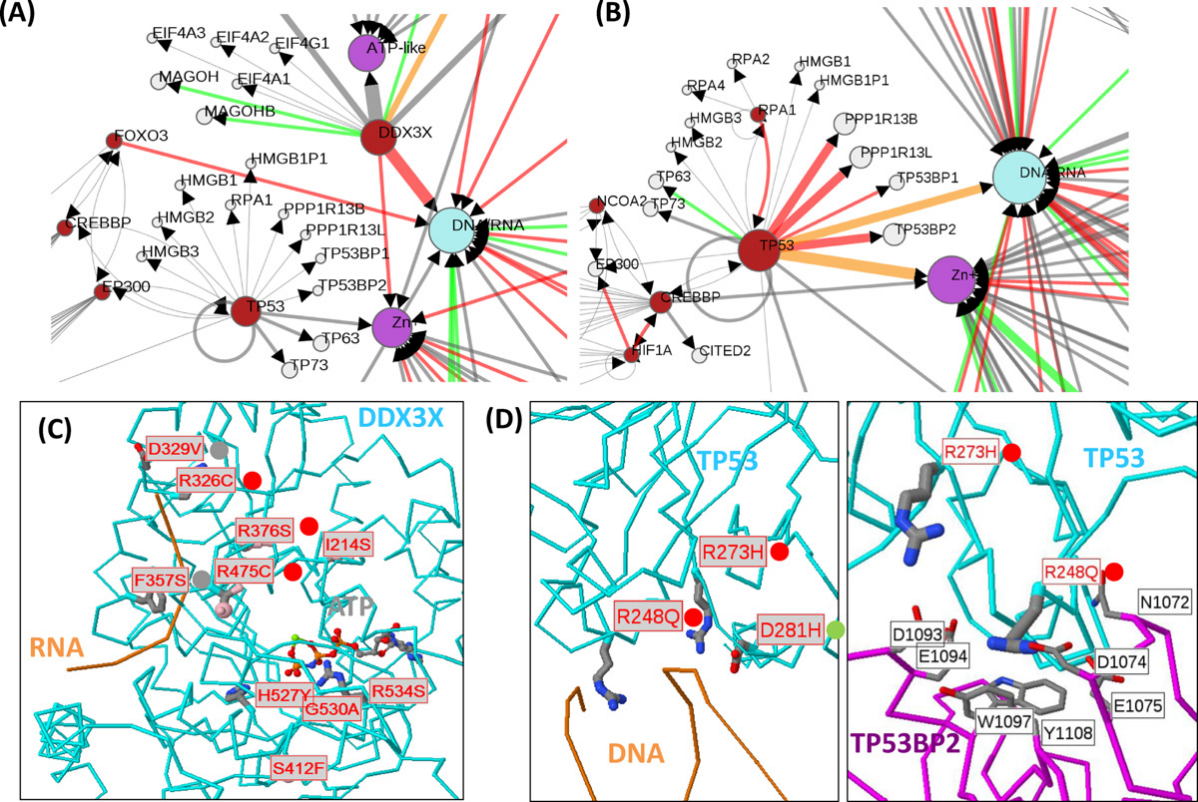
Variants in HTS cancer data sets. (**A**) Portions of the wider network of interactions involving proteins (red if mutated, grey if not), chemicals (magenta) and DNA/RNA (blue) affected by mutations identified after sequencing Medulloblastoma tumors (43) and Pancreatic cancer (**B**). The size of the red protein nodes is proportional to the number of variants contained within them, the size of chemical and DNA/RNA nodes is proportional to the number of sites predicted to interact with them, and the width of edges is proportional to the number of sites affecting them. Red edges are those where the effect of the mutations is predicted to diminish the interaction, green to enhance and orange where different mutations have opposite effects. (**C**) Structures of DDX3X showing Medulloblastoma mutations affecting DNA or ATP-binding, and (**D**) mutations in Pancreatic cancer affecting functional interactions of TP53 with DNA and TP53BP2. Networks and protein structures are displayed as described in Figure 3.

As known from the original analyses of the gene sets, the two samples differ substantially in the proteins that are mutated, but Mechismo also highlights differences in how proteins common to both sets are affected. For example, both cancers have roughly the same proportion of variants in the tumor suppressor TP53 (6/690 or 0.85% in Medulloblastoma; 18/2805 or 0.64% in Pancreatic cancer). However, whereas none of those in Medulloblastoma are predicted to have strong functional consequences (Figure 4A), in Pancreatic cancer, 4/18 are predicted to affect protein interactions, two to affect interactions with DNA and three to affect metal/zinc binding (Figure 4B and D). Naturally, the mutations in Medulloblastoma could affect overall protein structure, but the fact that none of them lie directly at functional interfaces suggests an overall difference in the role of TP53 in these cancers. TP53 is also the most functionally compromised protein in Pancreatic cancer followed by KRAS and SMAD4. In Medulloblastoma, the only strongly affected protein in terms of function is DDX3X for which 4/10 variants affect DNA-binding (all disabling) and 5/10 affect ATP-binding (Figure 4C); this protein is believed to be a prominent player in many patients with Medulloblastoma (43).

### Application to phosphoproteomics data sets

Proteomics-identified PTMs can also illuminate molecular function and disease and, like mutations, many are known to target biomolecular interfaces (e.g. (2)). Applying Mechismo to a data set of phosphorylation sites in *E. coli* (45) predicts 21 sites to enhance/diminish protein interactions and a significant proportion of sites to be in contact with small molecules, predominantly metabolites and their analogues (Supplementary Figure S3A; *P* < 0.001 for 24.8% compared to 9.6–16.1% in shuffled data; Supplementary Table S4). Some are known to regulate enzymatic function, such as Ser-113 in isocitrate dehydrogenase, and Ser-102 in phosphoglucosamine mutase (45), the latter predicted here using a structure at low (35%) sequence identity. For most proteins, no regulatory phosphorylation is known even though the sites are clearly at the active site (Supplementary Figure S3B). Note that 21/26 of these sites are in enzymes and are in contact with phosphate groups (either alone or as part of another molecule), raising possibilities that phosphorylated residues were not modelled in the structures or are reaction intermediates.

### Deleterious and neutral mutations

Many methods attempt to distinguish deleterious from neutral mutations using information about residue conservation and individual known/predicted structures (e.g. (16–18)), though few of these consider biomolecular interactions explicitly. The data used to assess these methods (large sets of disease-causing or neutral mutations) are also a useful means to validate our approach. Many deleterious mutations abolish protein function by disrupting overall structure rather than directly affecting molecular recognition events. However, the high proportion of disease mutations at interfaces that we observed above suggested that this information can help identify functional deleterious mutations.

**Figure 5.**
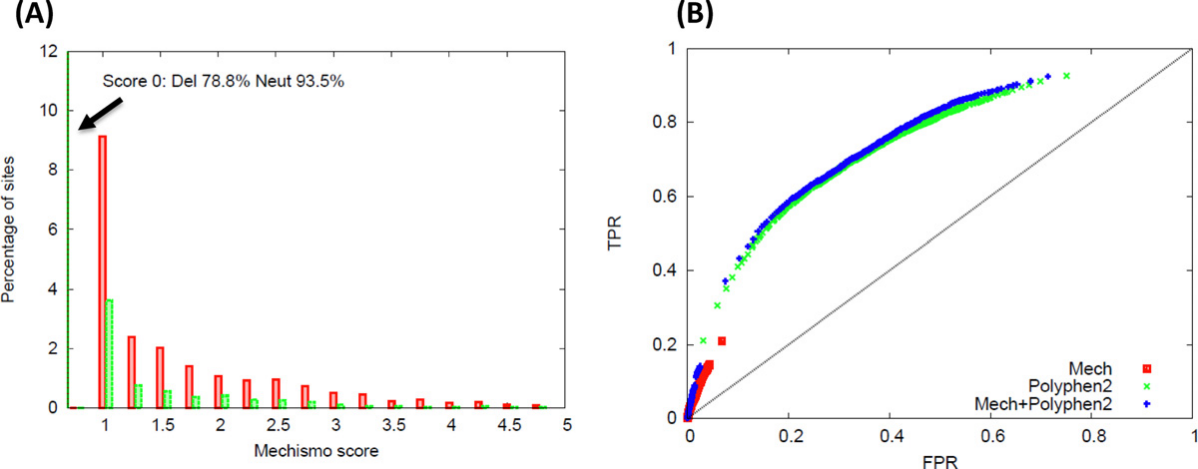
Mechismo as an aid to deleterious mutation predictions. (**A**) Distribution of Mechismo mutation scores for deleterious (red) and neutral (green) sites within a benchmark data set for deleterious site prediction. (**B**) ROC curve showing the effect of combining Mechismo and Polyphen2 scores to the same data set.

When running the method on a combined data set of 14 837 deleterious and 16 068 neutral mutations (53) there is a significant enrichment in protein, chemical and DNA/RNA interactions in deleterious mutations relative to neutral (*P* << 0.001) and this improves as a function of the Mechismo score (Figure 5A). This data set also shows a higher portion of disabling than enabling in both deleterious and neutral mutations, though there are a greater proportion of both enabling and disabling mutations in deleterious relative to neutral (Supplementary Figure S4). We expect both disabling and enabling to be generally deleterious to an organism as both ultimately affect a protein function such that it deviates from wild type.

It is important to emphasize that residues at functional sites make up only a small fraction of the total (13%). Mechismo by itself is thus a relatively poor overall predictor of deleteriousness (Figure 5B, Supplementary Figure S5) though the enrichment suggests that such specific functional information could potentially aid efforts to identify deleterious mutations in combination with other methods. When combining Mechismo scores (as normalized averages as done previously (20)) with those from PolyPhen2 (Figure 5B) and SIFTS (but not MutationAssessor or Condel) shows a very slight increase in AUC (Figure 5B, Supplementary Figure S5) and highlights a subset of mutations with a low FPR. We did not see any significant change in the results when only considering sites at interfaces (for all methods), which is likely to do with the fact that simply lying at an interface is a major determinant of whether a mutation will be deleterious or not.

There are also 435 known deleterious sites in the benchmark set that are at functional sites (Mechismo score ≥1; 74 have a score ≥2; 16 have ≥3) but which are not predicted to be deleterious by any of the methods, mostly owing to poor conservation, including several sites in TP53 (Supplementary Figure S6). A more detailed investigation into the possibility of combining this information with deleterious predictors will be published elsewhere.

## CONCLUSION

Despite many technical advances, it will take decades until structures of most interactions are available. This makes methods to extrapolate information from known structures to homologous proteins important for understanding biological mechanism. The mechanistic basis of why particular changes in proteins have the effect that they do is one of the next great challenges in biology and utterly requires a deeper integration of HTS and proteomics techniques with information related to protein 3D structures. When doing so, however, it is critical to exploit even weakly homologous structures, since this greatly increases the coverage of functional sites. This is particularly true for chemical or DNA/RNA sites as homologous proteins very often use a similar location to bind their ligands (63), and it is increasingly rare for a protein domain family to be entirely lacking in bound ligands. Protein interaction interfaces are less well covered by homologous structures, though a recent analysis suggested up to a third of known interactions have homologous structures (4) and that this proportion grows with each new complex structure solved.

Mechismo provides a rapid structural and mechanistic view of this mesmerizing volume of data, and will be useful for prioritizing modifications/variants, improving methods to predict deleterious mutations and understanding the biological or disease mechanisms of large sequencing or proteomics data sets.

## SUPPLEMENTARY DATA

Supplementary Data are available at NAR Online.

SUPPLEMENTARY DATA
