# Supplementary material for: Mechismo: predicting the mechanistic impact of mutations and modifications on molecular interactions
Source: Nucleic Acids Res. 2014 Nov 11;43(2):e10. doi: 10.1093/nar/gku1094 (PMC4333368; doi:10.1093/nar/gku1094)
Supplement: SUPPLEMENTARY DATA [file supp_43_2_e10__index.html]

Mechismo: predicting the mechanistic impact of mutations and modifications on molecular interactions — Mechismo: predicting the mechanistic impact of mutations and modifications on molecular interactions — SUPPLEMENTARY DATA 

# Mechismo: predicting the mechanistic impact of mutations and modifications on molecular interactions

## SUPPLEMENTARY DATA

**Files in this Data Supplement:**

- SUPPLEMENTARY DATA
