# Supplementary material for: Mechismo: predicting the mechanistic impact of mutations and modifications on molecular interactions
Source: Nucleic Acids Res. 2014 Nov 11;43(2):e10. doi: 10.1093/nar/gku1094 (PMC4333368; doi:10.1093/nar/gku1094)
Supplement: SUPPLEMENTARY DATA [file supp_gku1094_nar-02971-met-n-2014-File003.pdf]

Supplement for:

Mechismo: predicting the mechanistic impact of mutations and modifications on molecular interactions

Matthew J. Betts (1,2) Qianhao Lu (1,2), YingYing Jiang (1,2), Armin Drusko (1,2), Oliver Wichmann (1,2), Matthias Utz (1,2), Ilse Valtierra (1,2), Matthias Schlesner (3), Natalie Jaeger (3), David T. Jones (3), Stefan Pfister (3), Peter Lichter (3), Roland Eils (2,3), Reiner Siebert (4), Peer Bork (6), Gordana Apic (1,5), Anne-Claude Gavin (6), Robert B. Russell (1,2)*

1. Cell Networks, University of Heidelberg, Im Neuenheimer Feld 267, 69120 Heidelberg, Germany
2. Bioquant, University of Heidelberg, Im Neuenheimer Feld 267, 69120 Heidelberg, Germany
3. Deutsches Krebsforschungszentrum, Im Neuenheimer Feld 280, 69120 Heidelberg, Germany
4. Institut für Humangenetik, Universitätsklinikum Schleswig-Holstein, Christian-Albrechts-Universität zu Kiel, Arnold Heller Straße 3, 24105 Kiel, Germany
5. Cambridge Cell Networks Ltd, St John’s Innovation Centre, Cowley Road,CB3 0WS, Cambridge, UK.
6. EMBL, Meyerhofstrasse 1, 69117 Heidelberg, Germany

*Correspondence: robert.russell@bioquant.uni-heidelberg.de
Tel: +49 6221 54 513 62 FAX: +49 6221 54 514 86

Class	Total-chemicals	Total-instances	Most-common (het codes)
Organic	14120	27544	UNL BEN PHQ 5BU MTE
Drug-like	478	2102	ADN CHD PAR STU 017
Inorganic	133	649	BEF AF3 0QE CF0 C2O
Organomettalic	221	14837	PLP FMN SF4 SAH FES
Inorganic	80	140	FCO FNE FC6 CUB FCI
Sugar	128	9821	NAG MAN BMA GAL BGC
Zn+	1	8802	ZN
Mg++	1	8308	MG
Ca++	1	7184	CA
Mn++	1	2005	MN
Fe++	1	1079	FE
Cu+(+)	1	912	CU
Other-Metal	84	4277	NI CD CO FE2 HG
ATP-like	68	3758	ADP ATP ANP AMP ACP
GTP-like	55	1957	GDP GTP GNP 8OG DGT
UTP-like	28	729	UDP UMP U5P UPG DUP
CTP-like	24	507	DOC DCP CTP C5P CDP
TTP-like	23	421	THP TTP TMP TYD 2DT
NAD-like	6	1742	NAP NAD ZID 2NF NFD
FAD-like	6	1472	FAD FDA FAE 6FA FAA
Lys-like	41	1286	KCX LLP MLY M3L ALY
Trp-like	31	231	TRP DTR 0AF TYM FTR
Cys-like	26	1262	CSO CME OCS CSW CSX
Arg-like	26	251	ARG DAR HAR HMR 2MR
Tyr-like	20	221	TYR DTY NIY OMY OMZ
Phe-like	21	202	DPN PHE PHI MEA 4BF
Leu-like	18	317	LEU NLE DLE MLE MLU
Glu-like	16	880	PCA GLU DGL CGU FGA
Asp-like	15	223	ASP IAS DAS ACB PHD
Met-like	15	8049	MSE SAM FME MET CXM
Ala-like	13	507	CSD DAL ALA BAL MAA
Pro-like	13	273	HYP DPR PRO HZP N7P
His-like	11	172	HIC HIS DHI NEP MHS
Ile-like	11	46	ILE DIL IIL ILX IML
Asn-like	11	93	MEN ASN DSG AHB DMH
Gln-like	7	61	GLN DGN MEQ GNC NLQ
Thr-like	9	55	THR DTH ALO 2TL THC
Ser-like	9	184	DSN SER SAC SET HSE
Val-like	9	229	MVA DVA VAL FVA DIV
Gly-like	2	171	GLY OGA
pTyr-like	2	576	PTR TYS
pST-like	4	1061	SEP TPO D11 4TP
Heme-like	58	3814	HEM HEC SRM HEA HAS
CoA-like	46	609	COA ACO CAA MYA MLC
Steroid-like	55	157	DHT EST TES R18 AND
Fatty-acid	16	465	PLM MYR DAO DKA STE
Ion	15	282	OH O GD MO NH3
Plipid	37	225	FPP PGV LHG GPP DMA
Alkane	18	187	OCT D12 HEX D10 HP6
Lipid	17	596	BOG LMT BNG DMU UMQ

Table S1 List of chemical classes, with numbers of discrete chemicals and examples of the most common of each type.

Species	Total proteins	Prot-prot (%)	Prot-chem (%)	Prot-DNA/RNA (%)
H.sapiens	20213	10676 52.8	9334 46.2	2759 13.6
M.musculus	16670	9457 56.7	8282 49.7	2550 15.3
D.melanogaster	3231	1704 52.7	1563 48.4	590 18.3
C.elegans	3467	1590 45.9	1466 42.3	544 15.7
S.cerevisae	6621	2408 36.4	2163 32.7	770 11.6
E.coli	4433	2380 53.7	2032 45.8	401 9.0
B.subtilis	4185	1837 43.9	1684 40.2	340 8.1
M.pneumonia	687	286 41.6	240 34.9	131 19.1

Table S2 Breakdown of residues from each organism matched to structures interacting with other proteins, chemicals or DNA/RNA. The second number in each column are the percentages of the total covered by each.

Interactor	RhoA	Wt	R5Q	L69R	D76V
ARHGAP1	Pred	(+)		---	
	Obs	Positive		Negative	
ARHGAP20	Pred	(+)		++	
	Obs	Weak		Negative*	
ARHGDIA	Pred	(+)		---	
	Obs	Positive		NEGATIVE	
ARHGEF12	Pred	(+)	+/-	--	----
	Obs	Positive	Positive	Negative	Positive
ARHGEF25	Pred	(+)	++	---	-
	Obs	Positive	Positive	Negative	Moderate
MCF2L	Pred	(+)	+/-	-	
	Obs	Positive	Positive	Negative	
DIAPH1	Pred	(+)		---	
	Obs	Positive		NEGATIVE	

Table S3 Summary of Yeast two-hybrid results for RhoA and various mutants against a selection of known interaction partners. + or - symboles denote postive or negative and the number the strength relative to the observation for the wild-type. +/- denotes an ambiguous situation, and (+) denotes "predictions" for wild-type (wt) where all interactions have been observed previously. * denotes a Negative reported when the wt interaction was only very weak.

Dataset	Int	Original			Positions only				Proteins/positions				Surface positions only				Surface proteins/positions			
		T	C	%	T	C	%	P	T	C	%	P	T	C	%	P	T	C	%	P
Med	PP	675	63	9.33	680	60	8.82	0.072	678	56	8.26	0.06	663	51	7.69	0.044	667	56	8.4	0.064
Med	Nu	675	22	3.26	680	16	2.35	0.08	678	20	2.95	0.119	663	23	3.47	0.118	667	13	1.95	0.045
Med	Ch	675	64	9.48	680	53	7.79	0.042	678	37	5.46	0.002	663	55	8.3	0.057	667	46	6.9	0.018
PC	PP	1753	164	9.36	1752	149	8.5	0.032	1725	161	9.33	0.046	1720	150	8.72	0.038	1668	153	9.17	0.046
PC	Nu	1753	50	2.85	1752	53	3.03	0.076	1725	44	2.55	0.072	1720	47	2.73	0.08	1668	33	1.98	0.023
PC	Ch	1753	144	8.21	1752	115	6.56	0.032	1725	105	6.09	0.035	1720	117	6.81	0.039	1668	112	6.81	0.037
EcoPhos	PP	141	19	13.48	139	17	12.23	0.136	137	15	10.95	0.12	133	20	15.04	0.129	135	16	11.85	0.133
EcoPhos	Nu	141	10	7.09	139	7	5.04	0.156	137	7	5.11	0.16	133	6	4.51	0.14	135	10	7.41	0.184
EcoPhos	Ch	141	35	24.82	139	14	10.07	0.001	137	15	10.95	0.001	133	22	16.54	0.029	135	13	9.63	<0.001

.

Table S4 Enrichment and significance (Fischer test) calculations for the datasets discussed in the manuscript (Med = Medulloblastoma; PC = Pancreatic cancer; EcoPhos = E.coli phosphosites) in protein-protein (PP) protein-chemical (Ch) and protein-DNA/RNA (Nu) interfaces. T is the total sites in each set, C is the count at the particular interface, % is the percentage and P is the p-value for the difference between shuffles. Shuffled datasets are those in the columns labeled positions/proteins/surface. Percentages are colored yellow if significance is <0.05, orange if <0.01, and grey if the value is higher than lower (all other values are lower indicating enrichment whether significant or not).

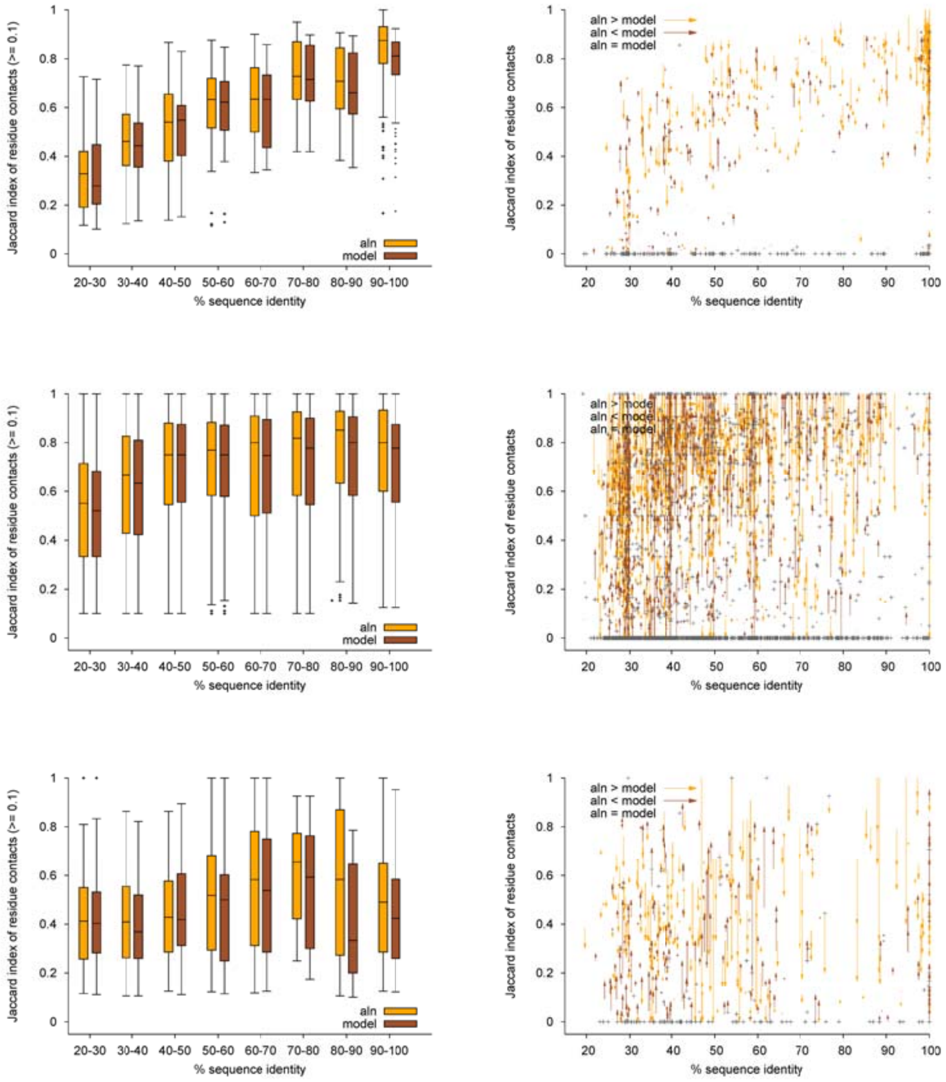

Figure S1 Box plots (Tukey; left) and difference plots showing the Jaccard fraction of contacts preserved when comparing an interface of known 3D structure to either a template/alignment (aln) or a constructed homology model (model) and a range of sequence identities between target and 3D structure, for protein-protein (top), protein-chemical (middle) and protein-nucleic acid (bottom) interfaces. The figures to the right show the difference (as vectors) between aln and model coloured and oriented according to which strategy is better for each data point.

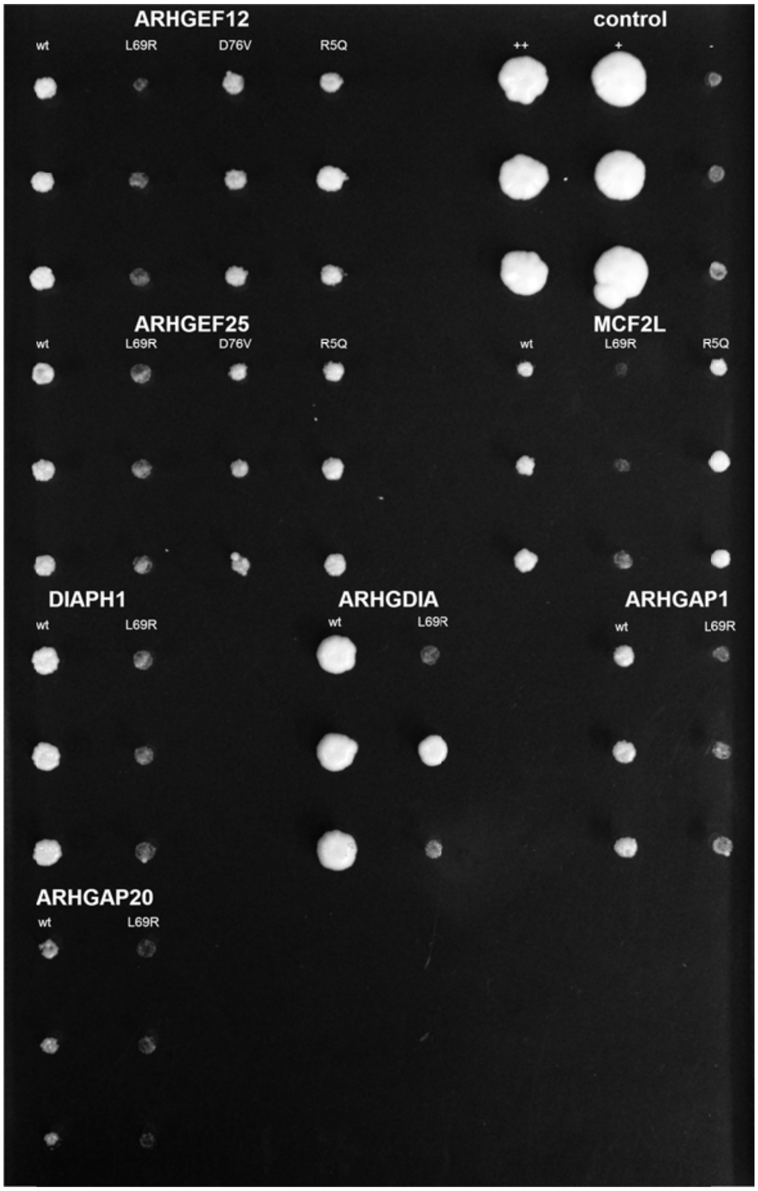

Figure S2 Yeast two-hybrid results for testing RhoA wild-type (wt) and mutants against a selection of known RhoA interactions (see Figure 3 and Table S3). For this image 3 individual colonies of each co-transformations were picked and suspended in 100 µl autoclaved saline. 10 µl of this suspension was transferred into 100 µl of selective growth medium (SC -Leu-Trp) and incubated at 30°C overnight. After that time cells were resuspended by vigorous shaking and spotted on assay plates (SC -Leu-Trp-His +10 mM 3AT). Phenotypes were assesed after 5 days.

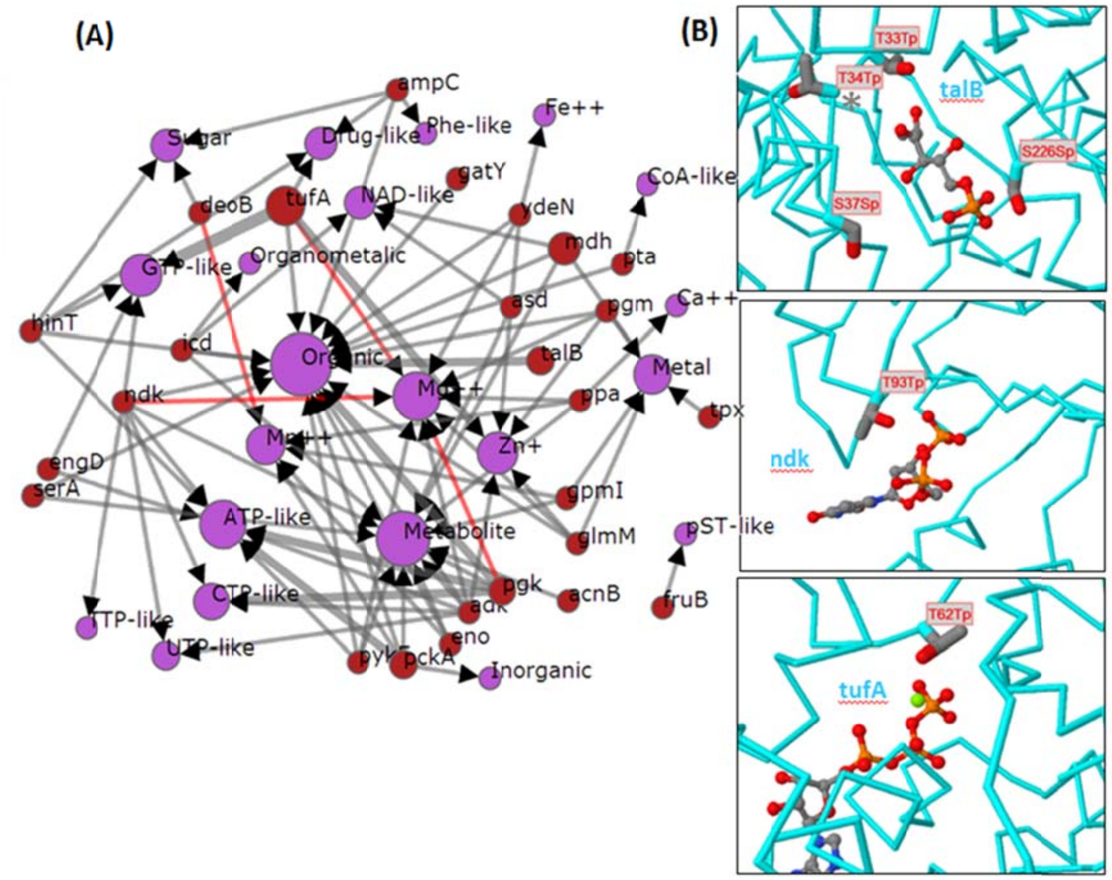

Figure S3 Studying the phosphoproteome of *E. coli*

A Networks of phosphoproteins in *E.coli* (45) interacting with small molecules. Structures in ***B*** show examples of sites in contact with phosphate groups. Other details are as for Figure 3. Note that the topmost sites in talB are likely one ambiguous site from the same peptide (*).

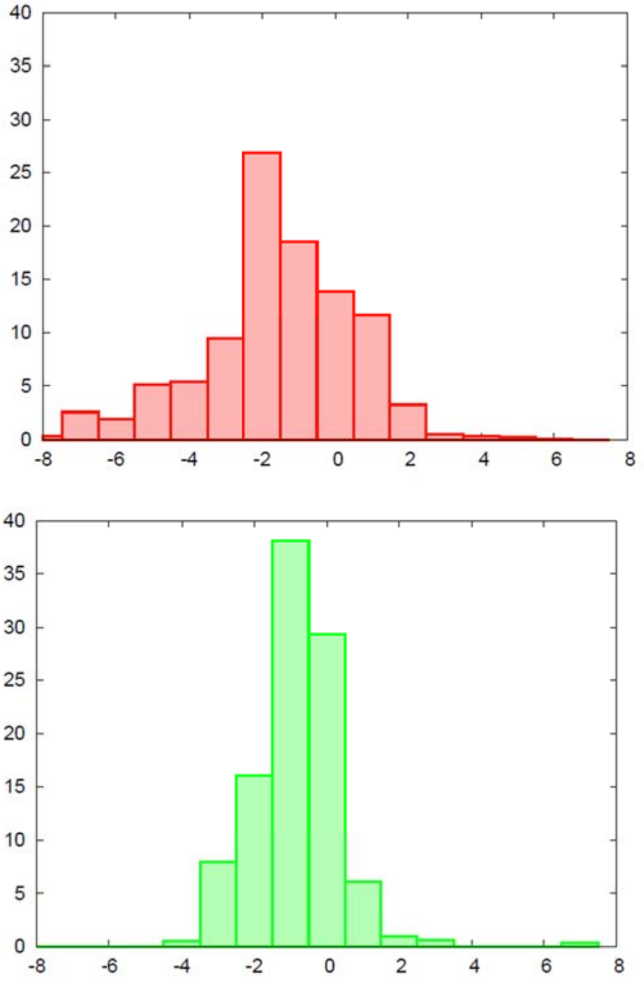

Figure S4 Distribution of protein-protein interaction interaction effect scores for 14,837 deleterious (top) and 16,068 neutral (bottom) mutations (Bendl et al, *PLoS Comput. Biol.*, **10**, e1003440, 2014).

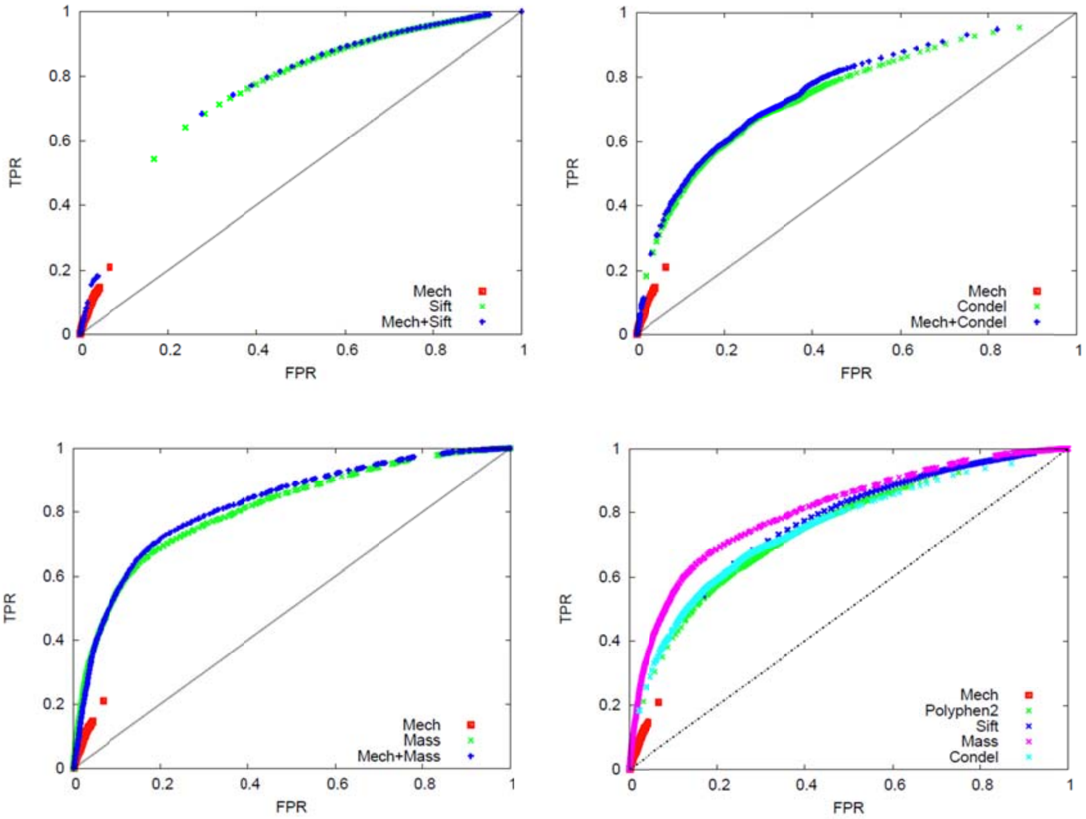

Figure S5 ROC curves assessing mechismo as a predictor of deleterious mutations. All plots are true positive rate (TPR) versus false positive rate (FPR).

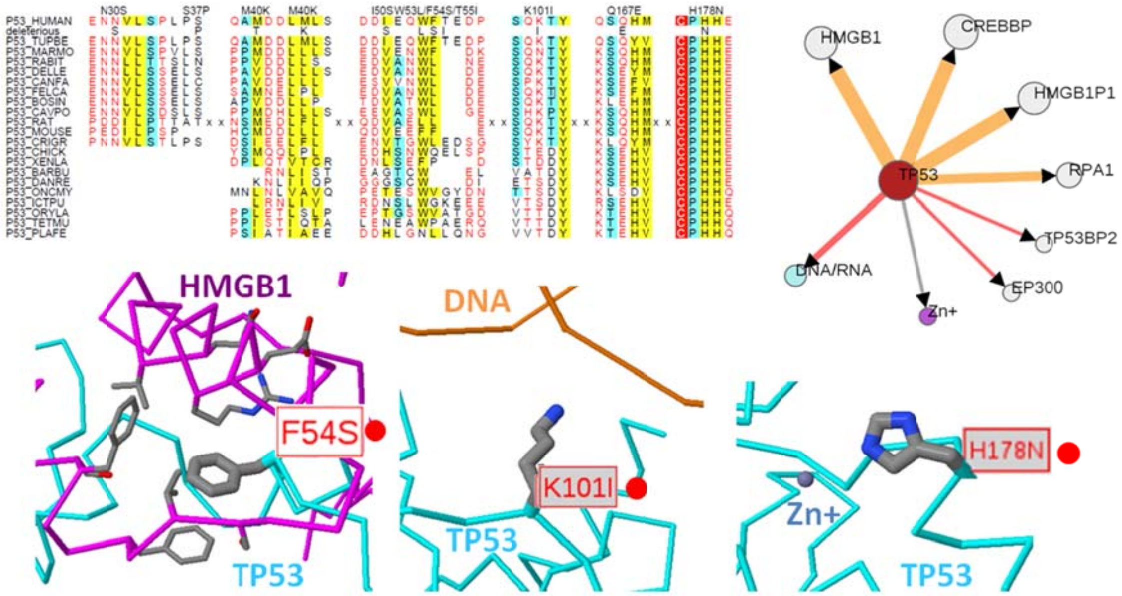

Figure S6 Deleterious sites found only by Mechismo. Selection of deleterious mutations in TP53 that are not predicted by any of the three predictors but which have clear functional consequences. The top left shows an Alscript (61) coloured alignment (62) of sequences within the regions around the mutations (labelled) with residues coloured according to residue properties if shared across more than 50% of the sequences in the alignment: hydrophobic, yellow background; polar, red colour, small, blue background; total conservation, boxed red. The network right of the alignment shows interacting molecules likely affected by these mutations, with red lines indicating predicted disabling and orange a mixture of disabling/enabling (i.e. multiple mutations affect the interaction differently). The structural diagrams below show the location of the mutations within the appropriate structures. TP53 is shown in cyan C-alpha trace, interaction partner HMGB1 in magenta, DNA in gold. Mutated residues are labelled in red, interacting residues in black. All are shown as ball-and-stick format colored by atom type (carbon grey, nitrogen blue, oxygen red).
